# The Hospitals and Surgeons of Paris

**Published:** 1843-10-14

**Authors:** 


*The Hospitals and Surgeons of Paris. An Historical and Statistical Account of the Civil Hospitals of Paris, with Miscellaneous information and Biographical Notices of some of the most eminent of the living Parisian Surgeons.* By F. Campbell Stewart, M. D. New York: J. & H. G. Langley. Philadelphia: Cary & Hart. 8vo. pp. 432.

In no country, as Dr. Stewart remarks in his Introduction, are greater pains taken to alleviate the condition of the great mass of the people than in France. The utter destitution and squalid poverty met with in Italy and other countries of Europe, are there scarcely ever seen. The public charities in France are on the most liberal footing, and the amount of annual relief afforded through the hospitals, and hôspices immense. Some idea of it may be gained, when we state that the average hospital population is twenty thousand, and that one hundred thousand poor persons receive pecuniary assistance, with the gratuitous distribution of clothes and provisions. The expenses of this noble charity are defrayed by the municipal government of Paris (an annual allowance of 5,200,000 fr.) by 10 per cent. on the gross receipts of all places of public amusements; from the pawnbroking establishments, and other sources. The total receipts in 1840, were three million four hundred thousand dollars. The outlay was two and a half millions of dollars, with all the economical precautions practised.

The whole number of public hospitals in Paris is thirty-six. These are under the government of a "General Council." They are admirably conducted. The wards are, in general, large and well ventilated, and the patients surrounded by every comfort. Many individuals of the middle ranks often resort to them in order to secure comforts and attendance which they could not command at home.

From this constant and immense population in the Parisian hospitals, the advantages and facilities offered the medical student are immense and unrivalled. No where can he acquire in the same space of time so much practical knowledge.

"The lectures on all the branches of science, delivered at the public schools, are gratuitous; and moreover as lecturing is one of the most common methods adopted for gaining notoriety and advancement, a number of young men will always be found engaged in repeating the lectures of the professors. These courses likewise are usually free, and the general, as well as special student, will find every avenue to learning laid open before him. The attainment of all kinds of knowledge is thus rendered comparatively facile, and in no other part of the world can general or professional studies be pursued to greater advantage, or at so little cost to the student, as in France." (*p.* 120.)

The condition of the medical profession in France is indisputably more elevated as a scientific body, than in this country. The tests of ability demanded are of the most rigid kind. With us, general attainments in science are rather prejudicial to the possessor, both in the eyes of the public and of his professional brethren. Here the profession of medicine hardly rises above a trade or calling. The men who in general are successful, would have been equally so in commerce, or in any other pursuit where a certain amount of acuteness and cleverness are the only or chief requisites. A skilful imitation of the tactics of the acknowledged charlatan, seems just now to be the order of the day. This consists in the adoption of the latest surgical or medical novelty, the immediate publication of all the experiments, and their dissemination over the country, by means of the medical journals, or by pamphlets. Whilst our remarks on the low standard of scientific attainments admit of but little modification, there are happily many exceptions to this professional charlatanism. Still its increase is alarming, and should, we think, be dealt with unsparingly. Whilst in France there has been a gradual elevation of the standard of professional acquirements, within the last few years, with us the reverse has occurred, and the deterioration in the general characterof the profession has been general and rapid.

"The professors of the various schools, receiving their salaries from the national treasury, there is but little jealousy among them, and small and contemptible means are never resorted to for the purpose of attracting scholars, by offering them undue inducements for the speedy and easy attainment of their degrees. Being wholly independent of the student, and receiving no remuneration from him, other than a small examination fee, professors are willing to do justice to the responsible positions which they occupy, and by a fair, but rigid examination, endeavour in all cases to ascertain the positive qualifications of candidates before they will consent to accord them licenses to practice." (*p.* 122.)

The work whose title heads this article, and which has led to the above hasty remarks, has afforded us much instruction and amusement. We have read it throughout with the greatest pleasure. It has served to recal scenes for a long while familiar to us, to refresh our memory on many important and interesting points, and to make us constantly regret that we had not enjoyed the advantage of such a work some years since, when pursuing our studies abroad. To the student about visiting Paris, it is indispensable. Time, trouble, and money will be saved to him by its possession. To the American Physician, it will give, besides a full account of the whole system of instruction in the Medical Metropolis, the police of the hospitals, valuable statistics regarding the mortality, &c.; the dietary arrangements; the standard classical medical works in the language; the best journals and their character; with correct, sprightly written accounts of the most distinguished French Surgeons; and all done in a modest, clever, and attractive manner. We cordially and strongly recommend this work to our readers, it fulfilling all the anticipations we indulged in when we announced it some time since.

The omission of the decimal weights and measures employed in France, since 1840, with their English equivalents, will be supplied doubtless in a subsequent edition, together with the correction of several typographical errors of proper names.

